# Investigation of 2H/1T/1T′ phase MoS_2_ optical nonlinearity

**DOI:** 10.1186/s11671-025-04321-8

**Published:** 2025-08-06

**Authors:** Hsuan-Sen Wang, Shih-Po Su, Yi-Hsuan Huang, Li-Wei Tu, Paritosh V. Wadekar, Hsiang-Chen Wang, Chao-Kuei Lee

**Affiliations:** 1 Department of Photonics, National Sun Yat-Sen University, Kaohsiung, 80424 Taiwan; 2 Department of Physics, National Sun Yat-Sen University, Kaohsiung, 80424 Taiwan; 3 Department of Mechanical Engineering, National Chung Cheng University, Chiayi, 62102 Taiwan

## Abstract

2H and 1T/1T′ molybdenum disulfide (MoS_2_) are typical phases that can be found in crystalline and thin film materials. In this work, by controlling the atmosphere during thin film chemical vapor deposition, 2H or 1T/1T′ phase MoS_2_ are grown separately. Additionally, by employing the Z-scan technique, the phase-dependent optical nonlinearity of MoS_2_ is observed and investigated. The 2H phase-dominated few-layered MoS_2_ shows clear reversed saturable absorption with a peak intensity reaching $3.3 \, \text{GW}/{\text{cm}}^{2}$ 3.78 GW/cm^2^, indicating extra higher-order nonlinear absorption. In contrast with the 2H phase, dominant single photon absorption is observed in the 1T/1T′ phase MoS_2_. In addition, the nonlinear refractive index (n_2_) of the two phases is characterized, exhibiting values of 1.82 × 10^–10^ cm^2^/W (1T/1T′ phase) and − 4.82 × 10^–10^ cm^2^/W (2H phase). This is the first time that the phase-dependent optical nonlinearity of MoS_2_ has been distinguished. Meanwhile, the proposed methodology not only provides information on the differences between the phases but also serves as a guide for determining suitable phases in specific applications.

## Introduction

In the last decade, two-dimensional (2D) materials, such as graphene, topological insulators, black phosphorus, transition metal dichalcogenides (TMDs), and Mxene, have attracted significant attention owing to their advantageous properties and potential in numerous applications [1–10]. 2D materials consist of layered structures that are weakly bonded between each layer by van der Waals forces, leading to variation in the band structure depending on the number of layers [11–18]. Among the different types of 2D materials, TMDs have beneficial optoelectronic properties. Compared with other TMDs, molybdenum disulfide (MoS_2_) has been widely studied because of its relative abundance on Earth. To prepare a single layer or few layers of MoS_2_, lithium is inserted into the bulk MoS_2_ as an intercalation reagent, and then the layers are exfoliated to obtain a single layer of MoS_2_, but a crucial drawback for this method is that a structural phase transformation occurs during Li intercalation [19, 20]. In addition, some special methods are generally used to process MoS_2_, such as spin coating, hydrothermal methods, and physical vapor deposition [21–26]. The MoS_2_ samples in this work are prepared by chemical vapor deposition (CVD).

To date, MoS_2_ has been applied not only in electronic devices but also in photonic devices [27–30]. Notably, the bandgap of few-layer MoS_2_ is suitable for interacting with wavelengths in the visible range, indicating that it has potential in solar cell and light-emitting diode applications. In terms of nonlinear optics, MoS_2_ has also been used in saturated absorbers and optical limiters [31–34], resulting from saturable absorption (SA) and reverse saturation absorption (RSA) behaviors, respectively. Monolayer MoS_2_ has a direct bandgap of approximately 1.8 eV, whereas few-layer MoS_2_ has a narrower indirect bandgap of 1.2 eV [35–38]. As a result, the variation in nonlinear absorption may be attributed to the change in bandgap energy with the number of MoS_2_ layers. Furthermore, various phases, such as 2H and 1T/1T′, may be found and even coexist during the preparation [39–42]. The different characteristics and behaviors of these phases have been reported. However, the corresponding phase-dependent optical nonlinearity, which is strongly related to its performance in applications, has not been clarified.

In this work, by employing a Z-scan technique, the optical nonlinearity of MoS_2_ in different phases was characterized. Compared with the saturation behavior of the 1T/1T′ phase MoS_2_, a nonlinear absorption transition from SA to RSA was observed with increasing excitation intensity in the 2H phase MoS_2_. Moreover, the corresponding nonlinear refractive index of the two phases was investigated.

## 2H, 1T/1T′ phase MoS_2_ thin film preparation

**Fig. 1:**
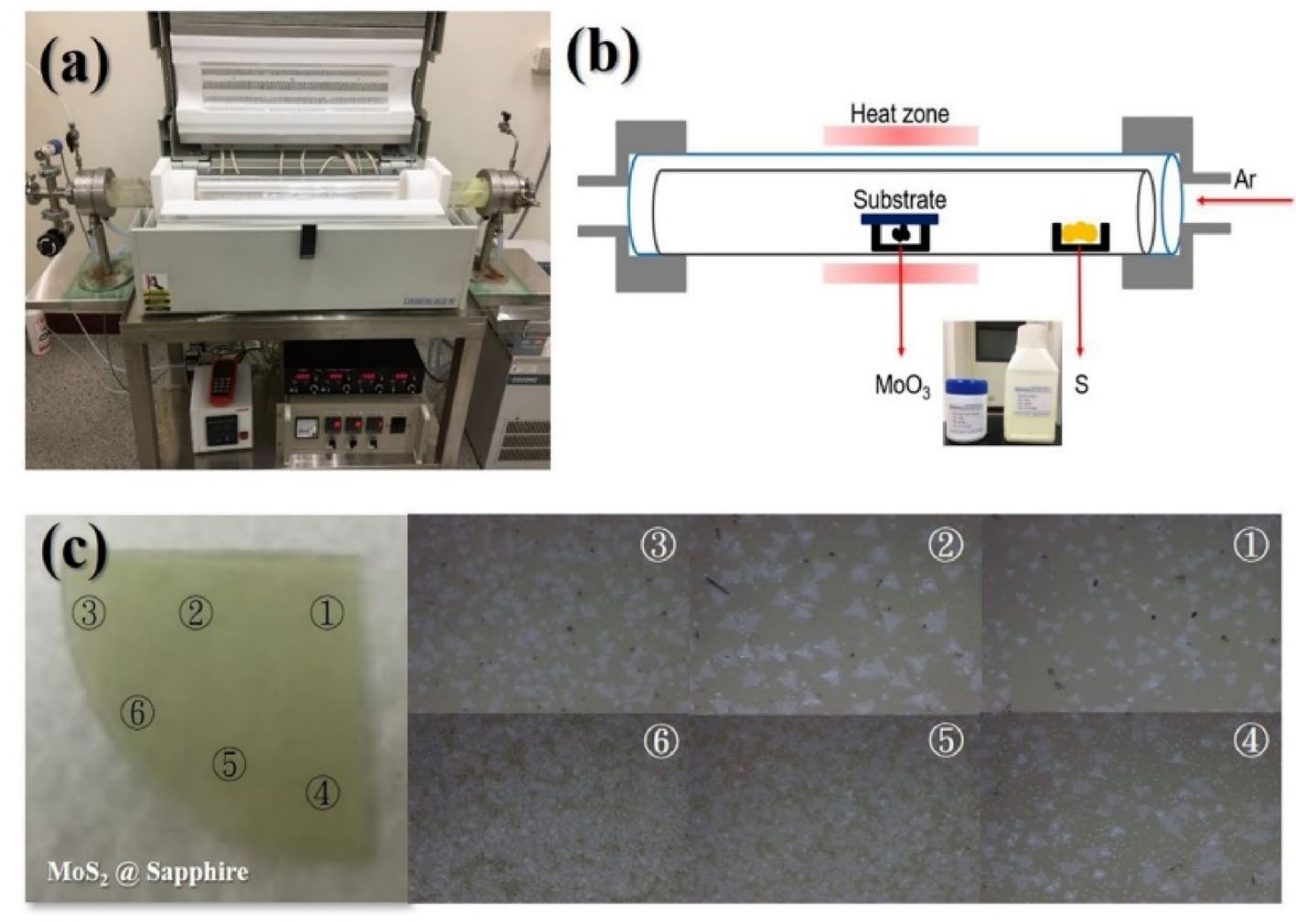
**a** Photograph of the three-zone furnace tube used in this experiment Lindberg/Blue HTF55347C. **b** Schematic of the experimental setup and positions of the precursors. **c** Area-coded position and optical microscope images of the sample

Figure 1 illustrates the experimental setup and the placement of the precursors used in the chemical vapor deposition (CVD) process for fabricating MoS_2_ thin films. Specifically, Fig. 1a presents a photograph of the three-zone furnace tube (Lindberg/Blue HTF55347C) used in the deposition experiment. The quartz tube in the furnace is clearly visible, showing where the substrates and precursors are positioned during growth. Figure 1b provides a schematic representation detailing the experimental configuration. Here, sulfur powder and MoO_3_ powder precursors are carefully placed inside the quartz tube, with sulfur positioned upstream (windward side), and MoO_3_ positioned at the center of the furnace, directly beneath the sapphire substrate. This strategic placement ensures precise control of vaporization rates and accurate phase formation of MoS_2_ on the substrate. Finally, Fig. 1c shows an area-coded schematic alongside corresponding optical microscope images of the grown sample, indicating distinct areas where the different MoS_2_ phases (2H and 1T/1T′) were successfully achieved. The MoS_2_ thin films in this work were grown on sapphire substrates using the chemical vapor deposition (CVD) method. The precursors included sulfur powder (99.98%, Echo Chemical Co., Miaoli County, Taiwan) and MoO_3_ powder (99.95%, Echo Chemical Co., Miaoli County, Taiwan). Similar work was reported by Rajesh et al. [43] in 2014, where MoS_2_ flakes were deposited on silicon substrates with oxide layers of 100 and 300 nm thickness. They immersed substrates into 5 mL of n-butyl lithium solution [44–50], a process carried out under an inert atmosphere (argon or nitrogen) at room temperature. After two hours of soaking, the samples were sequentially washed with n-hexane, deionized water, and isopropanol to remove organic residues and lithium ions. The samples were subsequently dried with nitrogen gas, and the 1T phase transformation was confirmed by Raman microscopy [51–54].

**Fig. 2:**
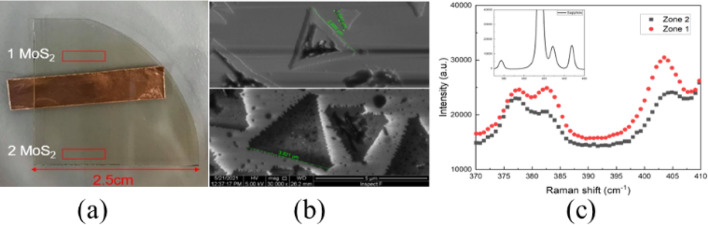
**a** Photographs of two areas comprising MoS_2_ flakes, 1 MoS_2_ hereafter refer to Zone 1 and 2 MoS_2_ hereafter refer to Zone 2, **b** SEM images of Zone 1Top and Zone 2 bottom, **c** Raman spectra of Zones 1 and 2; inset: Raman spectra of the sapphire substrate

Figure 2a shows a picture of the MoS_2_ thin film. Zones 1 and 2 are different areas grown on the sapphire substrate. Figure 2b shows scanning electron microscopy (SEM) images, which were used to identify and characterize flakes with few layers. The typical triangle shape MoS_2_ flakes were observed. By atomic force microscopy, the thicknesses of Zones 1 and 2 were determined to be approximately 1.74 and 1.75 nm, respectively. This indicates that the two areas have the same number of layers, see Fig. 2b. To determine the phase of Zones 1 and 2, Raman microscopy was performed. Figure 2c reveals the Raman spectrum of Zones 1 and 2. Compared with the Raman peaks of sapphire substrates, as shown in the inset, typical Raman peaks (382 and 404 cm^−1^) from MoS_2_ were observed, indicating the phases of MoS_2_. The 382 and 404 cm^−1^ signals are considered to be stronger in the 2H phase, which can be used to distinguish the 2H and 1T/1T′ phases. The results show that the 2H phase was dominant in Zone 2, whereas the 1T/1T′ phase was dominant in Zone 1[55].

## Optical nonlinearity measurement results and discussion

**Fig. 3:**
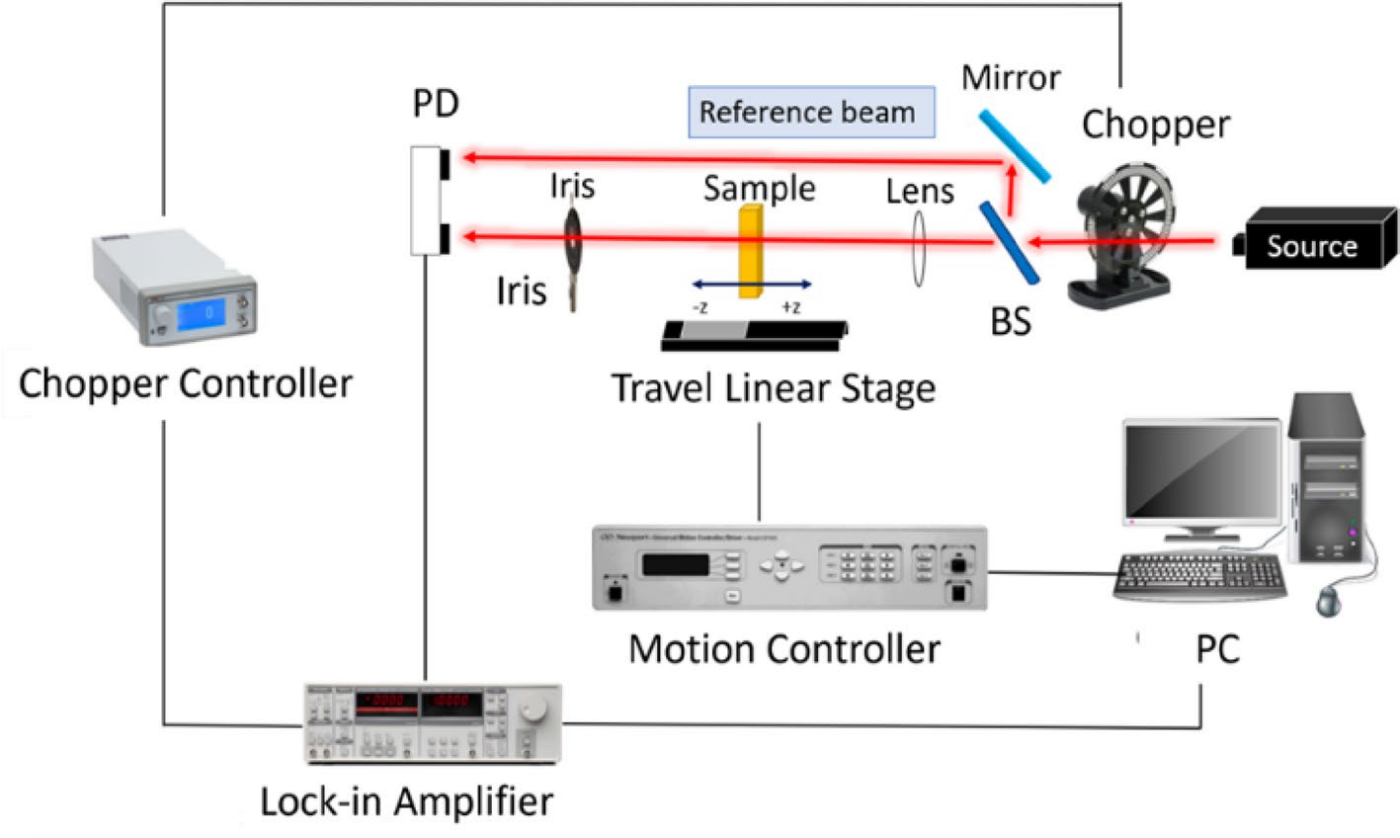
Experimental setup for Z-scans

After the 2H and 1T/1T′ phase MoS_2_ thin films were prepared, the optical nonlinearity of the two phases was studied. Among several nonlinear optical techniques employed to characterize nonlinear photonic materials, Z-scan techniques were conducted herein. The Z-scan system consisted of a Yb-doped fiber laser with a central wavelength, pulse width, and repetition rate of 1030 nm, 130 fs, and 27.9 MHz, respectively. The experimental setup of the Z-scan system is shown in Fig. 3. A combination of attenuators and neutral-density filters was used to adjust the input and output laser powers. A convex lens with a focal length of 30 cm was used to focus the laser beam, and the beam waist ω_0_ was approximately 39 μm. The calculated Rayleigh length, ZR = πω_0_^2^/λ, was approximately 0.45 cm. The sample was fixed on a motorized translation stage and oriented perpendicular to the input beam. A closed aperture was set behind the sample, and a convex lens was used to direct light into the photodetector. The Z-scan measurement for three-layer graphene was performed first, and the value of n_2_ was measured to be 1.15 × 10^–8^ cm^2^/W, confirming the accuracy of the system [56].

**Fig. 4:**
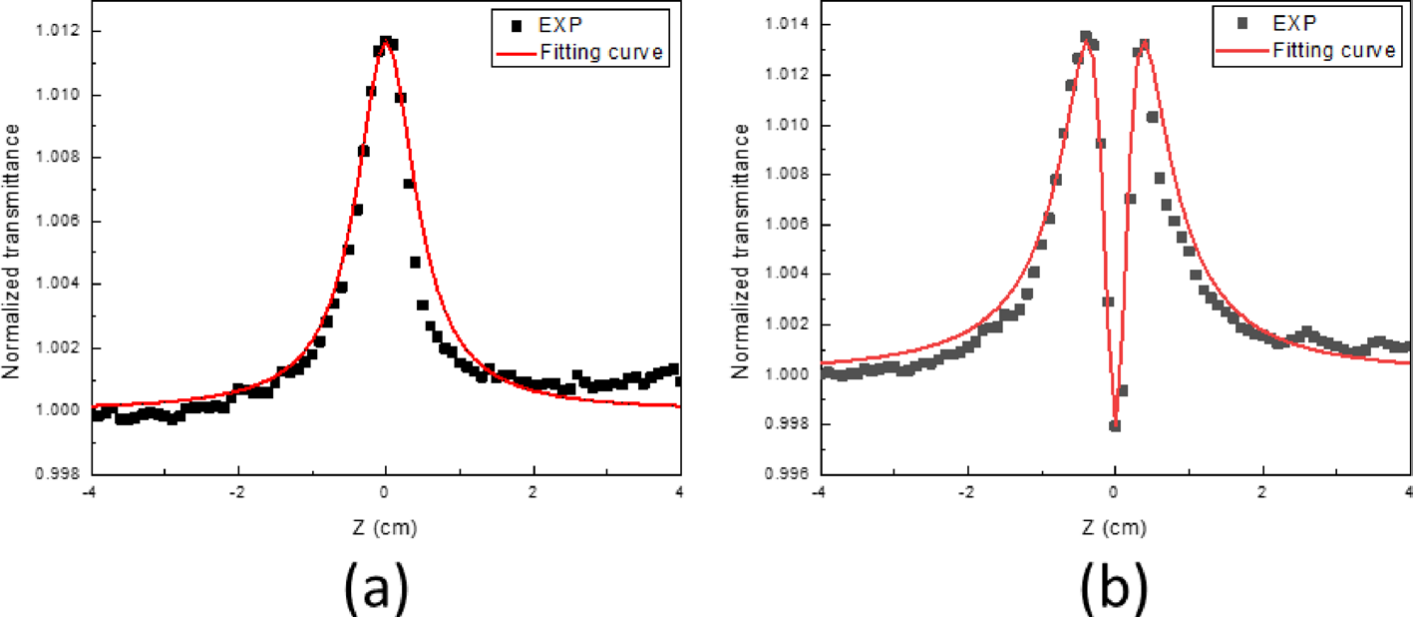
OA Z-scan at peak intensity I_0_ of 3.78 GW/cm^2^ and fitting results of Zone 1 **a** and Zone 2 **b**

Figure 4a shows the open aperture (OA) curve of Zone 1 at an excitation of 3.78 GW/cm^2^. The normalized nonlinear transmittance (NLT) first increased and then decreased along the Z-axis from − 40 to 40 mm. Next, the experimental results were initially simulated by considering only the SA from single photon absorption (SPA). The simulation equation is expressed as follows [57]:

1 $$T=\left[1-\frac{{\alpha }_{0}{I}_{s}L}{{I}_{s}+ {I}_{0}/(1+\frac{{Z}^{2}}{{Z}_{R}^{2}})} \right] /(1-{\alpha }_{0}L)$$

where T is the normalized transmission, L is the thickness of the sample, α_0_ is the linear absorption coefficient, I_0_ is the pulse peak intensity, I_s_ is the saturable intensity, Z_R_ = πω_0_^2^/λ is the Rayleigh distance, λ is the wavelength, and ω_0_ is the waist radius of the Gaussian beam. The simulation results are shown as the red line in Fig. 4a, revealing that the simulation agrees well with the experimental results. A saturation intensity of approximately 6.5 GW/cm^2^ was estimated accordingly. In contrast with Zone 1, the OA curve for Zone 2 exhibits different behavior (see Fig. 4b). This indicates that nonlinear absorption from SPA is not enough to depict this phenomenon, and higher-order nonlinearity should be included. Then, we accounted for two-photon absorption (TPA) in the simulation. The corrected transmission equation including TPA is expressed as follows [58]:

2 $$T\left(Z\right)=\frac{\text{ln}[1+\beta {I}_{0}{L}_{eff}/(1+({Z}^{2}/{Z}_{R}^{2}))]}{\beta {I}_{0}{L}_{eff}/(1+({Z}^{2}/{Z}_{R}^{2}))}$$

here T(z) is the normalized transmission, β is the two-photon absorption coefficient, I_0_ is the incident intensity, L_eff_ is the effective sample length, Z is the sample position along the beam propagation axis, and Z_R_ is the Rayleigh length. The simulation results are plotted as the red line in Fig. 4b, which is relatively consistent with the experiment in this case. The TPA coefficient β and saturation intensity I_s_ were estimated to be 0.018 cm/W and 3.9 GW/cm^2^, respectively. For clarity, the curves of Fig. 4a were also fitted using Eq. (2). Despite having nearly, the same value of Is from Eq. 1, the value of β is too small to characterize the TPA. This reveals that SPA may be the main contribution to the nonlinear absorption in Zone 1.

**Fig. 5:**
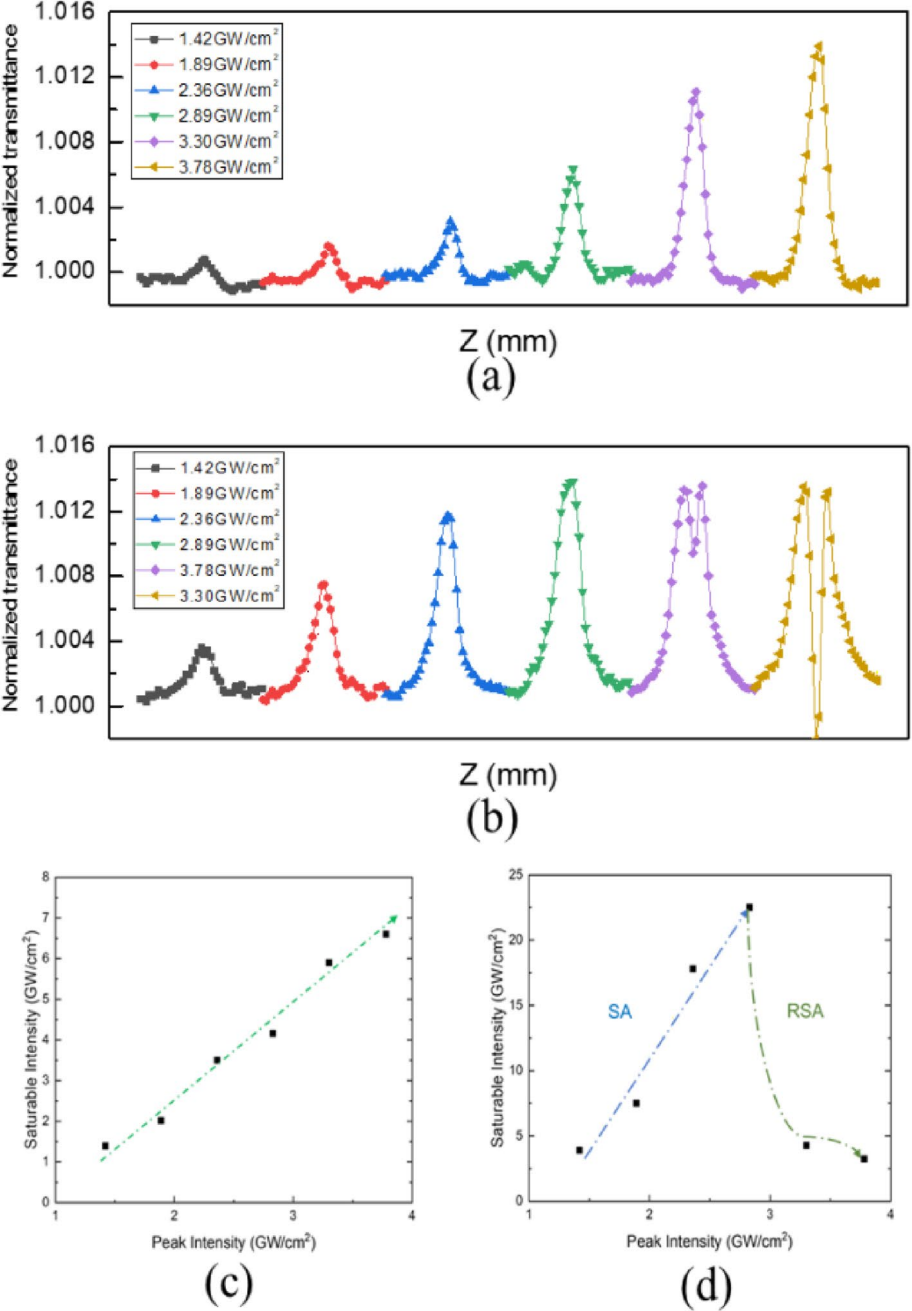
Power-dependent OA Z-scan from 1.42 to 3.78 GW/cm^2^ of Zone 1 **a** and Zone 2 **b**; saturable intensity versus peak intensity of Zone 1 **c**; saturable intensity versus peak intensity of Zone 2 **d**; **b** Power-dependent OA Z-scan for Zone 2 2H phase MoS_2_. Traces are shown for different peak intensities, clearly labeled in ascending order: 1.42, 1.89, 2.36, 2.83, 3.30, and 3.78 GW/cm^2^

Additionally, the power-dependent OA curves of Zones 1 and 2 and their analysis were characterized, see Fig. 5. Figure 5a shows the power-dependent OA curve for Zone 1. All the OA curves of Zone 1 show SA behavior, with the excitation peak intensity ranging from 1.42 to 3.78 GW/cm^2^. The corresponding power-dependent saturation intensity was plotted, as shown in Fig. 5c. The saturation intensity increases linearly with the excitation peak intensity. This can be attributed to the reduction in carrier relaxation time with increasing pump power, which leads to an increase in saturation intensity [59]. Furthermore, the power-dependent OA curve of Zone 2 was characterized (see Fig. 5b). Notably**,** in Fig. 5b, the trace corresponding to the highest peak intensity (3.78 GW/cm^2^) unexpectedly shows higher transmittance at the beam waist compared to the trace measured at 3.30 GW/cm^2^. This seemingly counterintuitive behavior results from the intricate balance between saturable absorption (SA) and two-photon absorption (TPA). At 3.78 GW/cm^2^, saturable absorption dominates locally, temporarily increasing the measured transmittance. In contrast, at 3.30 GW/cm^2^, the effect of TPA (which reduces transmittance) is relatively stronger, thus explaining why the lower intensity trace shows lower transmittance at the beam waist. The OA curve at low peak intensity shows typical SA behavior. That is, the normalized NLT increased at first and then decreased along the Z-axis. When the excitation peak intensity increased further, the normalized NLT still increased at first, but decreased as the sample approached the focal point, exhibiting a dip at the focal point. In addition, the behavior about decreasing as approaching the focal point become more serious as the peak intensity increased. This can be ascribed to the stronger contribution of TPA, together with the strengthening of the excitation peak intensity. Note that the two phases of MoS_2_ have the same layer number, suggesting that the thickness dependence was not a factor. Thus, the results can be attributed to phase-dependent properties.

The 2H phase has generally been recognized as the pure phase of MoS_2_ [60, 61]. In contrast, the 1T/1T′ phase contains vacancies. As a result, the band structure of 1T/1T′ phase MoS_2_ exhibits an extra intermediate state within the bandgap [55, 62]. From the Raman spectrum shown in Fig. 2c, the 1T/1T′ and 2H phases dominate in Zones 1 and 2, respectively. This may help interpret why the OA curves of Zone 1 all show SA, considering that the photon energy of the 1030 nm pulse is larger than the energy required to excite electrons to the intermediate state. On the contrary, pure MoS_2_ (2H phase) requires higher energy for electron transition, leading to TPA at high excitation peak intensities. The main debate has been whether MoS_2_ absorption originates from defects, or from the intermediate states within the materials. Herein, the results provide clear evidence for the origin of the SA. Additionally, Fig. 5d shows the values of Is obtained under various peak intensities in Zone 2 (2H phase MoS_2_), ranging from 1.42 to 3.78 GW/cm^2^. Furthermore, I_s_ increases from 3.9 to 22.5 GW/cm^2^ with increased excitation peak power. This may occur because the relaxation time diminishes with increasing excitation. However, the effective relaxation time becomes longer owing to the excited carrier that remains in the upper level longer, which is due to the extra transition from the valance band to the higher level, leading to a reduction in Is. The increase in β from 0.0135 to 0.0178 cm/GW and reduction in Is from 4.3 to 3.1 GW/cm^2^ with the increasing excitation peak intensity from 3.3 to 3.78 GW/cm^2^ are also consistent with the previous arguments. For Zone 1 (1T/1T′ phase MoS_2_), SA behavior was consistently observed at peak intensities up to 3.78 GW/cm^2^. Although higher intensity measurements were beyond the scope of this study, we anticipate that at substantially higher intensities, higher-order nonlinear absorption could become significant, potentially leading to a transition from SA to mixed SA-RSA behavior, similar to that observed in Zone 2. It is important to note that the saturation intensity (I_sat_) obtained from Z-scan measurements with femtosecond laser pulses is an effective value influenced by complex nonlinear optical interactions, including carrier dynamics, excited-state absorption, and multiphoton processes. Consequently, the effective I_sat_ may exhibit apparent intensity dependence, as observed in Fig. 5c, d. Additionally, when the numerically fitted I_sat_ exceeds the peak excitation intensity (I_0_), it reflects partial or incomplete saturation of absorption, indicative of nonlinear processes extending beyond single-photon absorption. Hence, the reported I_sat_ values should be interpreted as effective parameters for characterizing intensity-dependent nonlinear behaviors rather than intrinsic, fixed material constants. The observed differences in nonlinear absorption (NLA) between Zone 1 (1T/1T′-dominated MoS_2_) and Zone 2 (2H-dominated MoS_2_) primarily originate from their distinct electronic band structures and defect states. Zone 1, enriched with 1T/1T′ phases, exhibits saturable absorption (SA) predominantly due to single-photon absorption (SPA). This is because the 1T/1T′ phase possesses intermediate electronic states within the bandgap, enabling more efficient single-photon excitation pathways, thus readily leading to saturation at higher intensities. Conversely, Zone 2, dominated by the 2H phase, has fewer intermediate states, requiring significantly higher photon energies for electron excitation. Therefore, at lower intensities, Zone 2 initially demonstrates SA behavior. However, as intensity increases, two-photon absorption (TPA) becomes increasingly dominant, resulting in the transition from SA to reverse saturable absorption (RSA). Furthermore, defect states and vacancies prevalent in the 1T/1T′ phases facilitate quicker carrier relaxation, enhancing the SA effect. In contrast, the purer crystalline structure of 2H MoS_2_ reduces intermediate states and thus necessitates multiphoton processes at higher intensities, leading to the observed RSA behavior. Clearly distinguishing these phase-dependent nonlinear absorption mechanisms not only enhances fundamental understanding but also significantly informs potential optical applications. It should be noted that the maximum normalized transmittance value (~ 1.014) for Zone 1 (1T/1T′) coincidentally matches closely with the SA/RSA transition transmittance of Zone 2 (2H). Although this could potentially raise questions about instrumental or normalization influences, careful re-evaluation and cross-checking of our measurement methods confirmed that the observed trends are genuinely sample-dependent. Nevertheless, we acknowledge that additional experimental measurements at higher peak intensities for the 1T/1T′ samples are necessary to completely exclude the possibility of instrumental artifacts. Notably, the results shown in the Fig. 5 reveal the quality of our experiments. The close maximum for Zone 1 and 2 occurred at different excitation intensity, indicating the clear crossover behavior since the transmittance resulting from different absorption mechanism for phase of 1T/1T′ and 2H. Despite possible additional absorption for 1T/1T′ samples may exist for even higher intensity, unfortunately, additional experimental data at intensities higher than 3.78 GW/cm^2^ for the 1T/1T′ samples is un-available, due to the damage issue. However, our result still reasonably supports the proposed argument. The light source with smaller repetition rate for reducing the damage issue will be therefore required, and this will be considered as our research plan in the future.

**Fig. 6:**
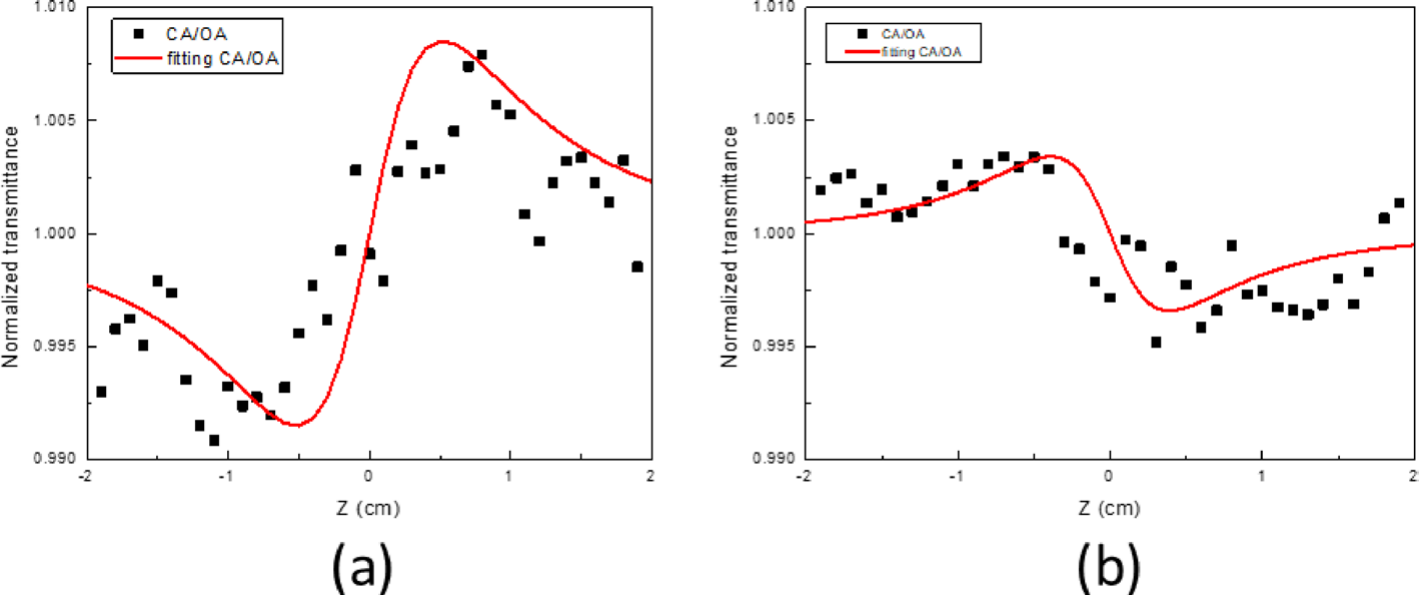
Closed aperture divided by OA Z-scan of Zone 1 Top **a** and Zone 2 bottom **b**

Additionally, the nonlinear refractive index of the sample can be further obtained through dividing the results of closed aperture by the results of open aperture, as shown in Fig. 6 (a) and (b). Figure 6(a-b) shows the normalized closed-aperture/open-aperture (CA/OA) Z-scan measurements and corresponding fits for Zones 1 and 2, obtained at a peak intensity of 3.78 GW/cm^2^. The fitting results are indicated as the red line according to the simulation equation expressed as below: [63]

3 $${T}_{close/open}=1+\frac{4(\frac{Z}{{Z}_{R}}){\Delta \varphi }_{0}}{\left[{\left(\frac{Z}{{Z}_{R}}\right)}^{2}+9\right]\left[{\left(\frac{Z}{{Z}_{R}}\right)}^{2}+1\right]}$$

where Δφ_0_ is the nonlinear phase shift at the focus (Z = 0), which is related to the nonlinear refractive index as n_2_ = Δφ_0_/(I_0_k_0_L_eff_). Here, I_0_ is the intensity of the laser beam, L_eff_ = [1 − exp(− αL)]/α, L is the thickness of the sample, α is the linear absorption coefficient, and k_0_ = 2π/λ is the wave vector. The CA/OA curve and the fitting curve of Zone 1 and Zone 2 at excitation peak intensity of 3.78 GW/cm^2^ were plotted and shown in Fig. 6a, b respectively. The retrieved nonlinear phase shift of Zones 1 and 2 were accordingly estimated with the value of 0.035 rad and − 0.017 rad. The corresponding nonlinear refractive index can be therefore calculated as 1.82 × 10^−10^ cm^2^/W and − 4.82 × 10^−10^ cm^2^/W respectively. This reveal that the laser with peak intensity of 3.78 GW/cm^2^ will experience self-focusing as passing through the Zone 1. In contrast, the beam will feel self-defocusing while meeting the Zone 2. Typically, there are several mechanisms, such as the dimension of the nanoflake or the variation of the nonlinear absorption, which the material will behave different value of nonlinear refractive index even the sign. In this work, the thickness and size are similar, leading to dominant of nonlinear absorption. Meanwhile, the higher order nonlinear absorption, such as TPA, will result in the sign change of the n_2_. As the above mentioned the difference between Zone 1 and 2, the 1T/1T′ phase MoS_2_ dominate in Zone 1 and saturable absorption due to SPA play the main role within the area. As for the Zone 2, the sign change of nonlinear refractive index is therefore expectable and reasonable since TPA is inevitable within the 2H MoS_2_ dominant. It is important to clarify that the negative nonlinear refractive index (n_2_) observed in Zone 2 (2H-dominated MoS_2_) does not directly originate from two-photon absorption (TPA) itself, as TPA is purely an absorptive nonlinear effect. Instead, the negative sign of n_2_ primarily arises from the excitation and subsequent generation of free carriers through multiphoton absorption processes (including TPA), which produce negative refractive index changes via free-carrier dispersion or thermal nonlinearities. When carriers are excited to conduction bands by femtosecond pulses, the resulting plasma or thermal-induced changes typically lead to negative refractive index shifts, causing the beam to self-defocus and yielding negative n_2_ values. Thus, the negative n_2_ measured in Zone 2 reflects these combined effects rather than being a direct result of TPA alone. Meanwhile, the positive n_2_ measured in Zone 1 (1T/1T′-dominated MoS_2_) results from resonant electronic transitions and different carrier relaxation dynamics, underscoring fundamental differences in carrier dynamics between the two phases. Although direct UV–Vis absorption measurements were not performed in this study, previous reports have indicated that the optical bandgap values for 2H and 1T/1T` phase MoS_2_ are approximately 1.2 eV and 1.8 eV, respectively [64–66]. These previously reported bandgap values align well with our observed nonlinear optical behaviors.

To date, MoS_2_ has been applied in various applications. In this work, the optical nonlinearity of the 2H and 1T/1T′ phase of MoS_2_ was characterized individually. In addition to identifying the contribution to optical nonlinearity, this work provides a way for adjusting the phase, indicating potential for new applications. For example, mixed MoS_2_ phases have been demonstrated in catalysts to increase conductivity and decrease free energy. This significantly enhances the hydrogen evolution reaction and simultaneously reduces the onset overpotential and Tafel profile. Therefore, this technology is expected to be applied in fuel cells to further improve their efficiency and performance [67]. Moreover, among various phases of MoS_2_, the 1T phase exhibits more effective photovoltaic properties because of its high absorption and low transfer resistance. These observations suggest that adjusting the phase is an effective way to tune and enhance performance. For example, reliable and cost-effective 1T and 2H miscible MoS_2_ nanobelts may replace expensive Pt counter electrodes in mass-produced dye-sensitized solar cells. These advances can help accelerate the development of solar cell technology and promote the application of renewable energy [68].

## Conclusion

In this work, 2H and 1T/1T′ phase MoS_2_ thin films were fabricated by controlling the atmosphere during CVD. 2H phase-dominated few-layered MoS_2_ shows clear RSA for peak intensities reaching 3.78 GW/cm^2^, indicating higher-order nonlinear absorption. In contrast with the 2H phase, SPA was dominant in the 1T/1T′ phase MoS_2_. This difference was attributed to the extra intermediate state within the 1T/1T′ phase MoS_2_. Meanwhile, the nonlinear refractive index (n_2_) of the two phases was characterized and fitted with values of 1.82 × 10^−10^ cm^2^/W (1T/1T′ phase) and − 4.82 × 10^−10^ cm^2^/W (2H phase). This is the first time that the phase-dependent optical nonlinearity of MoS_2_ has been distinguished. Moreover, the proposed methodology not only provides information on the differences between the phases but also serves as a guide for determining suitable phases in specific applications.

## Data Availability

The data presented in this study are available in this article upon considerable request to the corresponding author (H.-C.W.).
